# Transcriptomics of Post-Stroke Angiogenesis in the Aged Brain

**DOI:** 10.3389/fnagi.2014.00044

**Published:** 2014-03-18

**Authors:** Ana Maria Buga, Claudiu Margaritescu, Claus Juergen Scholz, Eugen Radu, Christine Zelenak, Aurel Popa-Wagner

**Affiliations:** ^1^Department of Psychiatry, University of Medicine Rostock, Rostock, Germany; ^2^Center of Clinical and Experimental Medicine, University of Medicine Craiova, Craiova, Romania; ^3^IZKF Lab for Microarray Applications, University of Würzburg, Würzburg, Germany; ^4^University of Medicine and Pharmacy Carol Davila, Bucharest, Romania; ^5^Molecular Oncology, Department of Medicine, Lady Davis Institute for Medical Research, McGill University, Montreal, QC, Canada

**Keywords:** aging, stroke, transcriptomics, angiogenesis

## Abstract

Despite the obvious clinical significance of post-stroke angiogenesis in aged subjects, a detailed transcriptomic analysis of post-stroke angiogenesis has not yet been undertaken in an aged experimental model. In this study, by combining stroke transcriptomics with immunohistochemistry in aged rats and post-stroke patients, we sought to identify an age-specific gene expression pattern that may characterize the angiogenic process after stroke. We found that both young and old infarcted rats initiated vigorous angiogenesis. However, the young rats had a higher vascular density by day 14 post-stroke. “New-for-stroke” genes that were linked to the increased vasculature density in young animals included *Angpt2, Angptl2, Angptl4, Cib1, Ccr2, Col4a2, Cxcl1, Lef1, Hhex, Lamc1, Nid2, Pcam1, Plod2, Runx3, Scpep1, S100a4, Tgfbi*, and *Wnt4*, which are required for sprouting angiogenesis, reconstruction of the basal lamina (BL), and the resolution phase. The vast majority of genes involved in sprouting angiogenesis (*Angpt2, Angptl4, Cib1, Col8a1, Nrp1, Pcam1, Pttg1ip, Rac2, Runx1, Tnp4, Wnt4*); reconstruction of a new BL (*Col4a2, Lamc1, Plod2*); or tube formation and maturation (*Angpt1, Gpc3, Igfbp7, Sparc, Tie2, Tnfsf10*), had however, a delayed upregulation in the aged rats. The angiogenic response in aged rats was further diminished by the persistent upregulation of “inflammatory” genes (*Cxcl12, Mmp8, Mmp12, Mmp14, Mpeg1, Tnfrsf1a, Tnfrsf1b*) and vigorous expression of genes required for the buildup of the fibrotic scar (*Cthrc1, Il6ra, Il13ar1, Il18, Mmp2, Rassf4, Tgfb1, Tgfbr2, Timp1*). Beyond this barrier, angiogenesis in the aged brains was similar to that in young brains. We also found that the aged human brain is capable of mounting a vigorous angiogenic response after stroke, which most likely reflects the remaining brain plasticity of the aged brain.

## Introduction

Recuperative therapeutic strategies for stroke are focused on revascularization, neuroprotection, and neuroregeneration, but most of the strategies that have been clinically tested failed to show benefit in humans. Post-stroke vascular remodeling is an essential event with crucial importance for neuroregeneration but unfortunately this process is still incompletely understood and therefore not exploited for therapeutic purposes (Masuda and Asahara, 2003; Hayashi et al., 2006; Caiado and Dias, 2012; Liman and Endres, 2012).

Aging is one of the most important risk factors for stroke (Barnett, 2002). Impaired neovascularization was described in elderly, but the effect of aging on angiogenesis and vascular remodeling after stroke has not been studied in detail. Previous studies from our group showed that, following insult to the brain, old rats are still capable of upregulating genes that are active during development, but the response is often blunted and temporarily uncoordinated (Buga et al., 2008).

Understanding mechanisms underlying angiogenesis and vascular remodeling after stroke in the elderly is crucial for developing new treatment strategies to improve the functional outcome after stroke in aged patients. Unfortunately, the molecular mechanisms regulating angiogenesis and vascular remodeling in aging brains are still poorly understood. Endothelial progenitor cells (EPCs) are likely to promote vasculogenesis after cerebral ischemia. Therefore, the regenerative potential of EPCs has been under intense investigation (Peichev et al., 2000). Many angiogenic factors, such as VEGF, IGFs, or FGFs, are involved in the mobilization of EPCs and increased levels of EPCs were correlated with increased plasma VEGF levels in stroke patients (Rafii et al., 2002). However, currently there is no effective and safe stem cell-based therapy for stroke (Lees et al., 2012).

Other studies have established that bone marrow-derived EPCs are present in the systemic circulation, and that they are able to differentiate into mature endothelial cells (EC) in the ischemic area, but the number of these cells is reduced by aging (Zhang et al., 2006; Mikirova et al., 2009). Previously, we have shown that cytokine-induced generation of bone marrow-derived EPCs can be enhanced by the administration of granulocyte-colony stimulating factor (G-CSF), and leads to improved functional outcome after stroke in aged rats (Popa-Wagner et al., 2010).

Few studies have investigated human post-stroke angiogenesis at the molecular level. Thus, Krupinski et al. (1994) noted active angiogenesis in the penumbral areas of patients who survived from several days to weeks after cerebral stroke, as well as a positive correlation between microvessel density and patient survival. In subsequent studies, the authors demonstrated an increased synthesis of angiogenic growth factors such as FGF-2, platelet-derived growth factor (PDGF), VEGF, and their receptors within hours of stroke that correlated with blood vessel growth in the penumbra (Krupinski et al., 1996, 1997).

The literature on gene expression profiles after stroke in humans also is limited. In this regard, Vikman and Edvinsson (2006) have shown similarities in gene expression profiles between human strokes and those in animal models, and reported new genes that support the dynamic changes that occur in the middle cerebral artery branches supplying the ischemic region. Also, promising results of blood genomic profiling in human stroke have been obtained in pilot studies (Moore et al., 2005; Tang et al., 2006; Tan et al., 2009). These results argue for the utility of pro-angiogenic therapies in stroke, given the potential effects consisting of increasing blood flow, decreasing infarct size, and supporting the restoration and recovery of neurovascular networks after ischemia (Liman and Endres, 2012).

Despite the obvious clinical significance of post-stroke angiogenesis in aged subjects, a detailed analysis of transcriptomics of post-stroke angiogenesis has not been done yet in an aged experimental model. By combining stroke transcriptomics with immunohistochemistry in aged rats and post-stroke patients, in this study we aimed at (i) identifying an age-specific gene expression pattern that may characterize the angiogenic process after stroke, (ii) exploring the potential of older animals to initiate regenerative processes following cerebral ischemia by supportive angiogenesis. This approach should allow us to identify new therapeutic targets that are crucial for enhancing neurorestoration after stroke in the elderly.

## Materials and Methods

### Animals

Thirty young (3–4 months of age) and 45 aged (19–20 months) male Sprague-Dawley rats, bred in-house, were used for the transcriptome analysis. The two age groups were divided randomly into 3- and 14-day post-stroke survival groups. In addition, 19 young (3–5 months of age) and 24 aged (18–20 months) male Sprague-Dawley rats were used as controls. Further 10 young and 15 aged post-stroke rats were used for the histological analysis. Body weights ranged from 290 to 360 g for the young rats and from 520 to 600 g for the aged rats. The group sizes for the aged rats were larger to compensate for the higher post-ischemic mortality rate (20%) as compared to young animals (12%) (Popa-Wagner et al., 1999, 2010). The rats were kept in standard cages in a temperature (22°C), humidity (40–60%), and light period (07.00–19.00 h) controlled environment and had free access to food and water. All experiments were approved by the University Animal Experimentation Ethics Board as meeting the ethical requirements of the German National Act on the Use of Experimental Animals.

### Reversible occlusion of the middle cerebral artery

Cerebral infarction was induced by transcranial interruption of blood flow by transiently lifting the middle cerebral artery with a tungsten hook as previously described (Popa-Wagner et al., 1999). Eighteen hours prior to surgery, rats were deprived of food to minimize variability in ischemic damage that can result from varying plasma glucose levels. Water remained available at all times. The right middle cerebral artery (MCAO) was lifted with a tungsten hook attached to a micromanipulator (Maerzhaeuser Precision Micro-manipulator Systems, Fine Science Tools). Both common carotid arteries were then occluded by tightening pre-positioned thread loops for 90 min. Throughout surgery, anesthesia was maintained by spontaneous inhalation of 1–1.5% halothane in a mixture of 75% nitrous oxide and 25% oxygen. Body temperature was controlled at 37°C by a Homeothermic Blanket System (Harvard Apparatus). The local changes in blood flow were monitored using a laser Doppler device (Perimed, Stockholm, Sweden), and blood gases were measured at several time points during ischemia. A decrease in the laser Doppler signal to <20% of control values was considered to indicate successful MCA occlusion. After 90 min, the common carotid arteries were re-opened. Surgery was performed under antiseptic conditions to minimize the risk of infection. Subsequent to survival times of 3 or 14 days, rats were deeply anesthetized with 2.5% halothane, 75% nitrous oxide, and 25% oxygen, and the blood removed by perfusion with neutral buffered saline. Brains were cut into 2 mm slices and the peri-infarcted area was microdissected under a microscope and stored at −70°C until use.

### RNA extraction and RNA quality control

After the tissue was homogenized, total RNA was extracted from microdissected tissue using TRIzol reagent (Invitrogen Life Technologies, Karlsruhe, Germany). Genomic DNA was removed using the RNeasy Plus kit (Qiagen).

### Microarray hybridization

The hybridization experiments were repeated two times on different occasions. Prior to sample preprocessing, RNA integrity of RNA pools was assessed with the RNA 6000 nano kit using the Bioanalyzer 2100 instrument (Agilent, Böblingen, Germany). RNA integrity numbers ranged between 6.5 and 8.2. Two hundred nanograms of each sample were processed with the whole transcript (WT) expression kit (Ambion, Darmstadt, Germany), i.e., subjected to RNA amplification via reverse transcription to double-stranded cDNA and subsequent *in vitro* transcription; this was followed by another round of reverse transcription yielding single-stranded DNA in sense orientation. Hybridization cocktails were produced after fragmentation and biotin labeling of target DNAs following the protocol of the GeneChip WT terminal labeling kit (Affymetrix, Santa Clara, CA, USA). Microarray hybridization to GeneChip Rat Gene 1.0 ST arrays (Affymetrix) was performed according to the manufacturer’s protocol using the Fluidics Station 450 with the program FS450_0007. CEL files from scanned microarrays were produced with the expression console (Affymetrix). Each array reflected the expression of 19–24 pooled animal samples. Since gene expression variance is notorious in aged animals, pooling of RNA from a large group of aged animals drastically reduces gene expression variance that is otherwise observed between individually hybridized animal samples. However, pooled design does not allow estimating within samples variation, which is one of the major limitations of our study. Nevertheless, the identification of differentially expressed genes is not adversely affected by pooling, and pooling has been recommended when fewer than three arrays are used in each condition (Kendziorski et al., 2005).

### Microarray evaluation

Consistently high quality microarray data was ensured by visual inspection of scanned images for hybridization artifacts and correspondence analysis (COA) of raw and normalized microarray data. Normalizations were performed with the Quantiles method (Bolstad et al., 2003); background correction and probe set summary were achieved with robust microarray average (RMA) (Irizarry et al., 2003). Differentially expressed genes were determined by comparing 3 days post-stroke vs. naive and 14 days post-stroke vs. naïve. These comparisons were done separately for young and aged animals. The false discovery rate (FDR) of differential expression for the described comparisons was estimated with an empirical Bayes methodology employing lognormal normal data modeling (Kendziorski et al., 2003) as previously described (Buga et al., 2012). Results were confined to probe sets belonging to gene ontologies (GO) related to angio- and vasculogenesis comprising GOs “angiogenesis” (GO:0001525), “vasculogenesis” (GO:0001570), “positive regulation of angiogenesis” (GO:0045766), “positive regulation of smooth muscle cell proliferation” (GO:0048661), and “blood vessel endothelial cell migration” (GO:0043534), which resulted in a total of 292 non-redundant probe sets. Expression values thereof were subjected to agglomerative hierarchical clustering and results were displayed as a heat map. The effect of stroke, post-stroke time, and age of rats was quantified by the Eigenvalues determined from a COA with 292 angio- and vasculogenesis probe sets. In brief, COA is an ordination method that performs its ordination simultaneously on column (sample) and row (probe set) scores such that dependencies between data points become evident. The COA visualization shows samples and probe sets in the same coordinate system, typically made up of the two most informative axes describing independent variation in the dataset. Due to the unsupervised nature of the COA, there is no predefined axis notation. All analyses were performed in R version 2.14.0^1^ along with Bioconductor^2^ packages affy (for data import and preprocessing), made4 (for COA and heatmap), and EBarrays (for detection of differential expression).

### Quantitative real-time PCR

All described genes were checked by RT-PCR. For quantitative real-time PCR (qPCR), we synthesized cDNA from large pools (*n* = 19–24) of total RNA with the high-capacity cDNA reverse transcription kit (Applied Biosystems, USA). The qPCR was performed in 96-well 0.1-ml thin-wall PCR plates (Applied Biosystems) in the Step One Plus System (Applied Biosystems). Each 20 μl reaction contained 10 μl iQ SYBR Green Master Mix (BioRad Laboratories, Hercules, CA, USA), 2 μl gene-specific forward and reverse primer mix (Qiagen, Alameda, CA, USA), and 8 μl pre-diluted cDNA. No template controls contained nuclease-free water instead. The cycling conditions were 3 min 95°C to activate iTaq DNA polymerase followed by 45 cycles with 30 s denaturation at 95°C, 30 s annealing at 58°C, and 30 s elongation at 72°C. At the end of the amplification cycles, melting curves were used to validate PCR product specificity. All samples were amplified in triplicate. Data were analyzed using the ΔΔCt method (Livak and Schmittgen, 2001). The expression levels of genes of interest were normalized to the average of expression level of the two housekeeping genes (hypoxanthine guanine phosphoribosyltransferase 1, HPRT1 and ribosomal protein 19, RPL 19) from the same sample. So the relative expression for a gene of interest was defined as the ratio of expression of the gene to that of the housekeeping gene. The fold change for a gene of interest was defined as the ratio of the relative expression in the ipsilateral hemisphere (stroke lesioned, peri-infarcted or PI) to that in the naive animals. Eurofinn, Germany provided all primers.

### Determination of the infarct volume

To assess the size of the infarct induced by transient focal ischemia, every 10th section was stained with NeuN, a marker of neuronal nuclei. In previous studies, we have found that the disappearance of NeuN is a reliable indicator of neuronal degeneration and death (Badan et al., 2003). Images of the stained sections were taken to cover the entire infarcted area, which was then calculated as the sum of partial areas using Image J analysis software. Integration of the resulting partial volumes gave the total volume of the ipsilateral hemisphere along with the total volume of the cortical infarct; lesion volume was then expressed as percent of the hemispheric volume.

### Immunohistochemistry of rat tissue

Sections (25 μm-thick) were cut on a freezing microtome and processed for immunohistochemistry as previously described (Popa-Wagner et al., 2007a). Briefly, after incubation with blocking solutions containing 3% donkey serum/10 mmol/L PBS/0.3% Tween 20, tissue sections were exposed overnight at 4°C either to mouse anti-NeuN (1:1000, Millipore, Germany) or mouse anti-rat CD31 (1:1000, AbD Serotec, Duesseldorf, Germany) diluted in PBS containing 3% normal donkey serum and 0.3% Tween 20. After washing in PBS containing 0.3% Tween, the signal was amplified utilizing an anti-mouse polymer-based secondary detection system (Histofine polymer-HRP, Nichirei, Japan) diluted 1:10 in PBS containing 1% normal goat serum and 0.3% Tween 20. After washing in PBS, sections were stained with tyramide–FITC.

For phenotyping, sections were incubated first with rabbit anti-laminin 5 (1:2000, Sigma, Munich, Germany) followed by mouse anti-P4HB monoclonal antibody (Novus Biological, UK). Cells and blood vessels were visualized by adding Cy3-conjugated donkey anti-rabbit IgG (H + L) (1:4000) or Cy2-conjugated donkey anti-mouse IgG (H + L) (1:3000).

For BrdU detection, free-floating sections were incubated in 2 M HCl at 37°C for 30 min, and rinsed in 0.1 M borate buffer (pH 8.5) at room temperature for 10 min. After neutralization, sections were incubated in blocking solution containing 10% goat serum, 0.3% Triton X-100, 0.2% gelatin in PBS overnight at 4°C, followed by rat anti-BrdU antibody (1:2000, Serotec, UK) at 4°C for 48 h. BrdU-positive cells were visualized by incubating with Cy5-conjugated donkey anti-rat IgG (H + L) (1:3000).

### Immunohistochemistry in human subjects

To analyze angiogenesis after ischemic stroke, formalin-fixed, paraffin-embedded archived brain tissue blocks containing both lesional and peri-lesional areas were selected from 15 stroke patients with a survival post-stroke time ranging from 12 h to 7 days. Two normal subjects were used as controls. Immediately after death, the bodies were refrigerated according to standard procedures and tissue samples were processed within 4–8 h from death. Death was attributed to complications such as massive edema and brain herniation with rostrocaudal deterioration, or cardiovascular arrhythmias. Written informed consent to autopsy was obtained for each patient from the relatives or their caregivers. Demographic data of patients and controls are given in Table 1.

**Table 1:** **Case demographics of ischemic stroke patients and controls**.

Patient	Age	Sex	Survival time
1	78	F	6 days
2	51	F	36 h
3	68	M	12 h
4	67	M	40 h
5	51	F	36 h
6	NA	M	20 h
7	66	M	30 h
8	77	F	7 days
9	68	F	2 days
10	82	M	12 h
11	77	F	5 days
12	64	F	28 h
13	74	M	12 h
14	65	M	2 days
15	83	F	–
16	68	M	*Control*
17	85	M	*Control*

A sequential methodology was utilized for NeuN/CD105 immunostaining. Briefly, after antigen retrieval, sections were incubated for 30 min in 1% hydrogen peroxide. After a blocking step of 1 h in 3% skim milk, the slides were incubated with the mouse anti-NeuN antibody (1:100; Millipore, Darmstadt, Germany) overnight at 4°C in a humidified chamber. The next day, the signal was amplified for 30 min utilizing an anti-mouse polymer-based secondary detection system (Nichirei-Histofine, Japan), and detected with DAB (Dako, Hamburg, Germany). In the second round of immunolabeling, the slides were incubated overnight with rabbit anti-CD105 antibody (1:100; LifeSpan Biosciences, USA). The next day, the antibody was amplified with a goat anti-rabbit biotinylated secondary antibody (Dako, Hamburg, Germany) together with AP-labeled streptavidin (ABC kit, Dako, Hamburg, Germany). The second signal was detected using Fast Red. After hematoxylin staining, the slides were coverslipped in a glycerol-based medium.

### Quantitation of microvascular density

Microvascular density was quantitated using the “hot spot” analysis covering 30% of the infarcted area. Briefly, hot-spots, i.e., regions with a high density of CD105-positive microvessels in humans and CD31-capillaries in rats, were identified using a 40× objective and were then counted using 20× objective, corresponding to a microscopic field of 0.7386 mm^2^. Counting was done by two independent observers and the results are expressed as means ± SD.

### Microscopy

For light microscopy, a Nikon Eclipse (Nikon, Duesseldorf, Germany) was used. Confocal microscopy images were acquired using a Zeiss LSM710 laser-scanning confocal system with spectral detection capabilities, and Zen 2010 software version 6.0 (Carl Zeiss Microscopy GmbH, Jena, Germany) was used for image acquisition and analysis. Excitation light was provided by 488, 543, and 634 nm laser lines; fluorescence emission was detected at 500–530 nm for FITC (green), 550–600 nm for Rhodamine (red), and 650–710 nm for Cy5 (blue) in separate tracks, using a confocal aperture of 1 Airy unit. Some of the images were acquired as *z*-stacks and 3D reconstruction was performed using a software algorithm (maximal projection).

### Enzyme-linked immunosorbent assay measurement of CD31

CD31 was measured via ELISA using homogenates from the per-infarcted area. Briefly, dissected tissue from the peri-infarction area, the corresponding contralateral area, and the sham control was homogenized in TRIzol reagent (Invitrogen, Germany). Sample preparation was performed according to the manufacturer’s protocol. Finally, the vacuum-dried protein pellet was dissolved in sample buffer containing 8 M urea, 2 M thiourea, 4% CHAPS, 65 mM DTT, and 40 mM Tris. Protein amounts were quantified by BCA protein assay (Pierce). ELISA plates (Santa Cruz, Heidelberg, Germany) were coated with 2 μg protein/well in coating buffer (1% deoxycholate, 10 mM sodium carbonate, 30 mM sodium bicarbonate, and 0.05% NaN_3_, pH 9.6) overnight at room temperature. The plate was washed three times with 1× PBS with 0.05% Tween 20 and incubated with blocking buffer (2% BSA in 1× PBS with 0.05% Tween 20) for 2 h at room temperature. For a negative control, wells were incubated with blocking buffer. Two replicate wells were prepared under each condition. After washing, the wells were incubated with a polyclonal mouse anti-CD31 (AbD Serotec, Duesseldorf) or rabbit anti-GAPDH antibody (New England Biolabs, Frankfurt) for 2 h, followed by washing and binding of secondary HRP-conjugated polymer (Histofine-HRP, Nichirei, Japan) for 2 h. The plate was washed three times, and then 100 μL of a 3,3′,5,5′-tetramethylbenzidine (TMB) substrate solution was added to each well. The plate was incubated at room temperature for 15–30 min and absorbance was measured at 450 nm with a universal microplate reader. Calibration was done using known amounts of CD31 standard.

### Dot blot analysis of CD31

CD31 was measured via a modified chemiluminescent ELISA using homogenates from the per-infarcted area. Two micrograms of protein were applied to a PVDF membrane using a vacuum dot blot apparatus (Hoeffer Scientific). From this step on, the procedures were performed in the well, much like an ELISA assay (i.e., without dismantling the dot blot apparatus). The wells were incubated with blocking buffer containing 2% BSA in TBS for 1 h at room temperature while being gently shaken. After washing, the wells were incubated with a polyclonal mouse anti-CD31 (AbD Serotec, Duesseldorf) or rabbit anti-GAPDH antibody (New England Biolabs, Frankfurt) for 2 h, followed by washing and binding of secondary alkaline phosphatase-conjugated polymer (Histofine-HRP, Nichirei, Japan) for 2 h. Finally the antigen–antibody complex was visualized by exposing the chemo-luminescent signal to X-ray film.

### Statistical analysis

The main effects of age, time, and manipulation (stroke vs. sham), as well as interactions, for the infarct volume, ELISA measurements and microvascular density in animal models were evaluated by ANOVA followed by Tukey *post hoc* analyses using SPSS software (SPSS Inc., Chicago, IL, USA). The level of significance was set at *p* ≤ 0.05, two-tailed test. For human samples, data were analyzed by using Student’s *t*-test. A *p* value of <0.05 was considered to be statistically significant. The relationship between metric data and histological data was assessed using Pearson’s product moment correlation (Pearson’s *r*).

## Results

**Figure 1:**
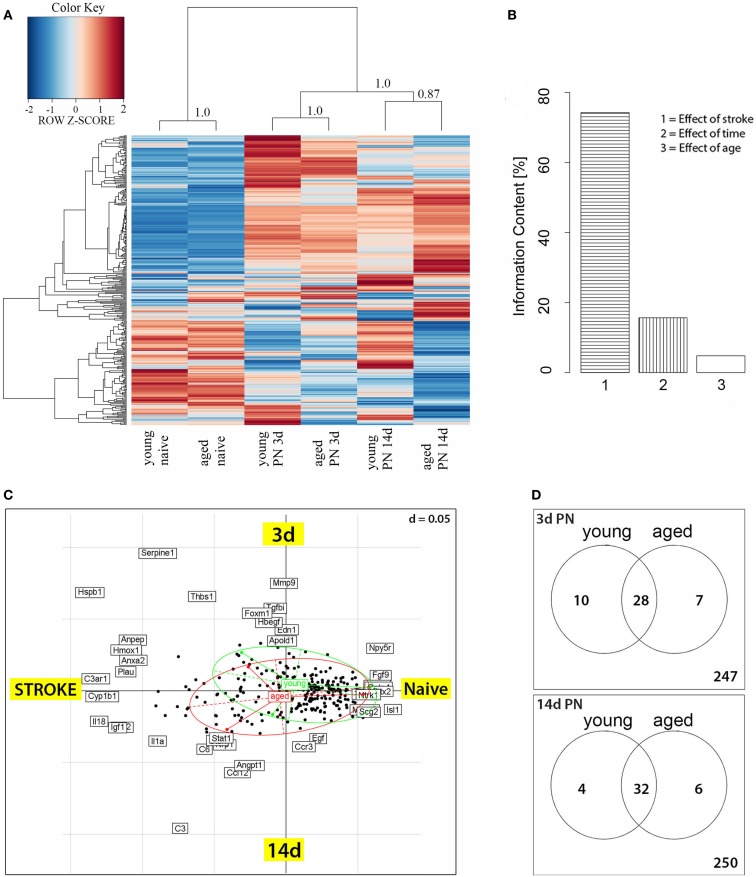
**(A)** Heatmap of genes differentially expressed between post-stroke and naïve animals. Scaled expression values of all 292 differentially expressed genes are shown for each group with deep blue being the lowest and dark red the highest expression level. The depicted dendrograms cluster samples (top) and genes (left) employing average agglomeration and Euclidian distance measure. Cluster branch stability was tested with the R-package “pvclust”. Approximately unbiased *p*-values calculated by multiscale bootstrap resampling are shown at cluster branches. **(B)** Correspondence analysis of differentially expressed genes and samples grouped by animal age. The panel depicts the Eigenvalues of the correspondence analysis and shows that the major factors contributing to the variance of stroketomics analysis were stroke (75%), post-stroke time (15%), and age (10%). **(C)** Sources of variability were stroke and post-stroke time, which formed the coordinates. The graph shows the distribution of transcripts (black dotes) as a function of treatment (stroke) and post-stroke time. Samples from young (green ellipse) and aged (red ellipse) animals show an extensive overlap as well as some difference in their post-stroke response. **(D)** Venn diagrams for 3 and 14 days post-stroke penumbra (PN) showing genes that were up- or downregulated exclusively in old or young rats, or in both age groups.

The dendrogram showed that relative expression values clearly distinguished naïve rats from their post-stroke littermates (Figure 1A). Within the group of infarcted rats, samples clustered according to the time following experimentally induced MCA occlusion. The left-hand dendrogram subdivided expression levels into two major groups; the larger one consisted of those probe sets whose expression was generally higher in post-stroke animals than in naïve ones, whereas the smaller group contained transcript clusters with reduced expression. In addition to these major groups, there was a group with a less consistent pattern of expression, i.e., those genes were weakly expressed in untreated animals, strongly expressed at 3 days after stroke, and then downregulated at 14 days. Finally, closer inspection of the heat map revealed that the age of rats also influenced gene expression levels, i.e., at day 3 the pattern of expression was similar in the two age groups but then became very dissimilar by day 14 post-stroke. For further examination of data, we performed a COA for those genes that were differentially expressed. By this method, we concluded that the major difference in gene expression between naive and post-stroke samples was caused by lesioning (effect of stroke), which accounted for 75% of the information content. Further, the post-stroke time accounted for 15% of the information content while age accounted for a surprisingly small proportion, <10% (Figure 1B). Further, in Figure 1C, we can nevertheless distinguish the age effect shown by the green circle for young and the red circle for aged animals. The effect of lesioning is here given as Axis 1, and the effect of the post-stroke time as Axis 2.

### Age-specific regulation of gene expression after stroke (Venn diagrams)

Of the 292 angio- and vasculogenesis probe sets, 59 were found to be differentially expressed in at least one of the comparison (Figure 1D). At day 3 post-stroke, 17 probe sets displayed an age-specific expression, thereof 10 in young and 7 in aged rats (Figure 1D); 28 probe sets are differentially expressed in both age groups. By day 14 after stroke, a total of 10 probe sets displayed an age-specific expression, 4 in the young group and 6 in the aged group, while 32 probe sets were shared by both age groups. The genes showing an age-specific expression are presented in detail below (Table 2).

**Table 2:** **New or age-related angiogenesis and vascular remodeling-related genes after stroke verified by RT-PCR**.

Sequence code	Gene symbol	Function	Young 3 days		Young 14 days		EP	Aged 3 days		Aged 14 days		EP
								
			FC	SD	FC	SD		FC	SD	FC	SD	
**INFLAMMATION-INDUCED VASCULAR REMODELING**												
10872361	*Bai2*	*De novo* angiogenesis inhibitor	−2.9	0.23	1.33	0.01		−2.70	0.14	−1.34	0.11	
10736697	Ccl2*	EC; monocyte chemoattractant protein; angiogenic	23.3	1.56	5.72	0.5	D	20.9	0.56	7.72	0.33	D
10771655	*Cxcl10*	Leukocyte recruitment via Tnfa signaling	2.90	0.51	6.36	0.43	D	3.65	0.09	9.00	1.1	D
10858165	*Cxcl12*	Chemoattractant, EPCs and SMC; promotes angiogenesis	2.07	0.21	3.06	0.17	A	2.81	0.15	6.15	0.35	A
10767373	*Cxcr4*	Chemoattractant EPC; promotes angiogenesis	13.7	0,22	3.81	0.54	D	9.37	0.33	4.68	0.28	D
10908896	Ets1*	TF, early stage; neovascularization	4.3	0.05	2.33	0.22	D	6.10	1.14	2.72	0.29	D
10907913	*Mmp8*	Macrophage collagenase; BM remodeling via Tnfa	6.3	0.1	3.34	0.09	D	4.34	0.14	8.01	0.34	A
10907869	Mmp12*	Fibroblasts collagenase (COL1)	12.3	0.23	3.54	0.1	B	14.0	0.45	7.59	0.33	D
10780205	Mmp14*	Collagenase; BL remodeling	4.7	0.46	5.07	0.70	A	7.72	0,90	11.2	0,59	A
10714103	*Mpeg1*	Inflammatory mediator; macrophages	4.6	0.31	4.15	0.34	A	3.90	0.22	9.14	0.44	C
10858967	*Tnfrsf1a*	Inflammatory mediator; apoptosis	3.2	0.02	3.33	0.04	A	3.78	0.17	6.29	0.07	A
10881424	*Tnfrsf1b*	Attenuates inflammation	4.6	0.25	4.16	0.20	A	4.41	0.14	8.28	0.10	A
**SPROUTING ANGIOGENESIS**												
10789291	*Angpt2*	Promotes the dissociation of pericytes	8.2	0.48	3.55	0.19	D	4.37	0.48	7.17	0.27	A
10835654	*Angptl2*	Macrophages; pro-angiogenic	4,3	0.21	3.32	0.15	A	2.44	0.11	3.99	0.2	A
10901166	*Angptl4*	EC; pro-angiogenic	4.5	0.2	1.90	0.24	B	2.48	0.4	8.51	0.02	A
10723032	Cib1*	Modulator of EC proliferation and migration	7.8	0.78	1.69	0.09	B	3.46	0.14	8.01	0.00	A
10750685	*Col8a1*	VSMC; fibroblasts and SMC proliferation via Tgfb	16.3	0.26	1.60	0.2	B	12.70	1.58	8.05	0.88	D
10914614	Ccr2*	Chemoattractant EPC; promotes angiogenesis	4.1	0.19	1.49	0.07	B	2.28	0.09	2.48	0.11	A
10775900	*Cxcl1*	Chemoattractant EPC; promotes angiogenesis	95.	6.77	8.25	1.97	D	22.0	4.16	19.1	1.15	A
10940663	Eng**	EC; pro-angiogenic via TGFb signaling	5.6	0.19	1.84	0.15	B	3.24	0.14	6.20	0.14	A
10715100	Hhex**	TF; promotes vasculogenesis via VEGF	6.1	0.32	2.29	0.26	D	4.29	0.27	3.12	0.07	D
10907689	*Itga5*	BV stabilization	3.6	0.22	1.60	0.06	B	2.88	0.11	1.90	0.09	B
10819052	*Lef1*	EC proliferation and migration	4.1	0.49	1.60	0.13	B	2.19	0.06	2.38	0.05	A
10808959	*Nrp1*		2.5	0.48	1.33	0.22	NC	2.81	0.26	5.72	1.02	A
10940659	*Pecam1*	EC marker; angiogenesis	5.5	0.48	1.65	0.08	B	2.89	0.18	3.38	0.21	A
10832299	*Pttg1ip*	EC proliferation and migration	6.4	0.45	2.59	0.27	D	4.28	0.25	2.90	0.11	D
10905316	*Rac2*	EPC polarity and proliferation	5.2	0.11	3.25	0.11	A	5.27	0.31	7.80	0.26	A
10753222	*Runx1*	EPC; pro-angiogenic	3.3	0.21	2.30	0.17	A	2.15	0.44	4.63	0.2	A
10758645	*Sh2b3*	EC adhesion and migration	4.0	0.32	2.23	0.16	A	3.78	0.28	2.65	0.5	A
10781022	*Sox7*	TF; arterial development; pro-angiogenic	4.9	0.77	1.64	0.23	B	5.97	0.95	1.36	0.28	B
10797726	*Tpm3*	EC migration	3.6	0.23	2.44	0.15	A	3.24	0.19	3.19	0.3	A
10790712	*Tpm4*	EC migration	3.7	0.21	1.12	0.07	B	3.74	0.22	3.38	0.09	A
10751048	*Tagln*	VSMC proliferation and differentiation	10.8	0.58	1.82	0.23	B	21.1	0.49	2.21	0.17	B
10873021	*Wnt4*	VSMC proliferation and migration	2.29	0.05	2.70	0.19	A	1.10	0.01	6.20	0.14	C
**RECONSTRUCTION OF BASAL LAMINA**												
10889339	*Adam17*	EC; ECM and vascular remodeling via *Tnfa* and *Il6ra*	2.66	0.2	1.83	0.1	B	4.45	0.3	2.70	0.5	A
10792997	Col4a2**	Reconstruction of a new BM	23	1.34	1.49	0.01	B	5.36	0.5	3.41	0.01	A
10929766	*Col6a3*	Reconstruction of a new BM	21.5	2.71	18.34	3.11	A	24.3	1.24	22.0	6.2	A
10928761	*Fn1*	BV stabilization	4.5	0.23	2.39	0.2	A	3.32	0.3	3.69	0.45	A
10768668	*Lamc1*	Reconstruction of a new BM	4.6	0.09	1.77	0.13	B	1.57	0.14	4.27	0.02	C
10782387	*Nid2*	Reconstruction of a new BM	3.6	0.29	2.20	0.63	A	1.57	0.32	2.77	0.91	C
10912255	Plod2**	Fibroblasts; collagen maturation; BM maturation	3.8	0.22	1.80	0.05	B	1.57	0.03	3.66	0.08	C
10757461	*Plod3*	Reconstruction of a new BM via COL4 and COL6	2.0	0.20	2.29	0.12	A	2.80	0.11	1.65	0.01	B
**TUBE FORMATION AND BV MATURATION**												
10903529	*Angpt1*	PVC; BV maturation/stabilization	2.7	0.51	3.78	0.61	A	2.48	0.4	8.51	0.61	A
10853559	*Col1a2*	Adventitial fibroblasts; aneurysms	2.2	0.09	3.9	0.09	A	1.59	0.13	4.32	0.10	C
10891780	*Fbln5*	EC; vascular remodeling; BV elasticity	10.7	1.5	2.03	0.03	D	8.66	0.5	2.60	0.15	D
10702913	*Fndc1*	BV stabilization	1.34	0.15	3.94	0.17	C	1.13	0.09	3.91	0.11	C
10939764	Gpc3*	Co-receptor for Fgf9; vascular development	1.74	0.00	3.18	0.49	A	5.38	0.64	6.16	0.62	A
10772169	*Igfbp7*	Vascular remodeling; lumen formation	1.79	0.13	2.84	0.10	C	1.49	0.06	4.37	0.45	C
10739721	*Itgb4*	Vascular integrity; BV maturation	3.65	0.1	2.97	0.28	A	2.74	0.55	2.96	0.3	A
10817711	*Notch2*	Pro-angiogenic via VEGFR2	1.7	0.10	2.30	0.02	C	1.36	0.12	2.45	0.08	C
10760971	*Pcolce*	Fibroblasts; vascular remodeling; lumen formation	1.34	0.2	3.84	0.1	C	1.52	0.3	5.78	0.31	C
10802040	*Pdgfrb*	Pericytes; BV stabilization	2.24	0.04	2.26	0.06	A	1.68	0.10	2.95	0.10	C
10753222	*Runx3*	Negative regulator of EPC	9.82	0.5	10.58	0.2	A	4.28	0.1	1.32	0.1	B
10746139	*Scpep1*	SMCs; elastogenesis and BV maturation	7.78	0.01	7.04	0.61	A	8.17	0.62	6.09	0.27	A
10817057	*S100a4*	SMC proliferation and migration; aneurysms	16.2	2.3	17.49	2.02	A	9.78	1.2	8.16	1.03	A
10742802	SPARC**	Pericytes recruitment; BV stabilization	1.97	0.12	4.84	0.51	C	2.49	0.13	8.42	0.55	C
10879278	*Tie1*	EC; modulates Angpt1/Tie2 signaling	2.52	0.05	1.69	0.11	B	2.10	0.06	1.67	0.09	B
10869946	*Tie2*	Sprouting angiogenesis via Angpt2	8.23	0.48	3.55	0.19	D	4.17	0.48	7.17	0.27	A
10814528	*Tnfsf10*	EC; pro-apoptotic	1.80	0.20	2.37	0.16	C	−1.15	0.40	9.01	0.22	C
	*Vegfc*		1.05	0.13	2.99	0.2	C	1.66	0.23	1.14	0.11	NC
10921772	Vegfa**	Sprouting angiogenesi and tube formation	1.15	0.09	2.40	0.07	C	1.34	0.22	1.09	0.05	NC
**BUILDUP OF FIBROTIC TISSUE**												
10737532	*Col1a1*	Fibrotic scar via Tgfb; aneurysm	6.7	0.65	16.88	0.78	C	10.5	1.41	45.4	1.06	C
10923052	*Col3a1*	Adventitial fibroblasts; aneurysms	16.0	0.91	57.82	0.32	C	32.8	2.44	237	7.44	C
10896263	Cthrc1*	Fibroblasts and SMC; fibrosis via Tgfb	13.0	1.47	1.80	0.02	B	11.4	0.22	10.8	1.46	A
10824530	Il6ra*	Promotes vascular leakage	1.90	0.15	1.90	0.60	NC	1.58	0.66	3.55	0.52	C
10813007	*Il6st*	Promotes vascular leakage	1.73	0.08	2.91	0.11	C	1.43	0.12	3.34	0.23	C
10754983	*Il13ra1*	Inhibits MMP secretion by fibroblasts; fibrosis	3.76	0.17	3.14	0.12	A	3.3	0.20	6.73	0.21	A
10909874	*Il18*	Microglia; pro-fibrotic via fibroblasts proliferation	4.17	0.24	1.77	0.04	B	3.66	0.22	7.59	0.44	C
10809540	*Mmp2*	Fibroblasts to myofibroblasts conversion	2.88	0.32	2.7	0.62	A	5.08	1.02	14.5	6.41	A
10749663	*P4hb*	Fibroblasts; collagen synthesis; fibrosis	2.34	0.29	2.08	0.07	A	2.39	0.66	2.84	0.19	A
10776459	*Pdgfra*	Fibrosis via TNFalpha and TGFbeta	1.74	0.05	2.06	0.03	C	1.38	0.02	3.15	0.03	C
10864874	*Rassf4*	Mesenchymal cells; fibrosis	6.25	0.1	12.24	1.46	A	4.73	0.86	23.3	2.9	C
10927842	*Stat1*	Promotes vascular leakage via Il6r	2.01	0.01	2.55	0.02	A	1.57	0.05	3.53	0.09	C
10893067	*Stat2*	Promotes vascular leakage via Il6r	1.59	0.04	1.67	0.06	NC	1.37	0.02	2.48	0.12	C
10868923	*Tgfbr1*	Fibroblasts and SMA proliferation; fibrosis	8.29	1.11	14.15	0.66	A	9.39	0.81	12.1	0.09	A
10920745	Tgfbr2*	Fibroblasts, SMC proliferation; fibrosis	5.06	0.44	3.80	0.40	A	6.87	0.71	10.1	0.46	A
10705213	*Tgfb1*	Fibroblasts and SMA proliferation; fibrosis	6.64	0.78	16.57	0.81	C	5.97	0.36	58.8	0.78	C
10797138	*Tgfbi*	Fibroblasts and SMA proliferation; inhibition	3.30	0.21	1.41	0.08	B	1.27	0.12	1.57	0.13	NC
10865094	*Wnt5b*	EC proliferation and migration	1.39	0.13	3.29	0.28	C	3.13	1.27	5.10	0.73	A
10936482	*Timp1*	Inhibits MMPs	40.6	2.34	8.53	1.11	D	41.5	3.64	31.1	1.18	A

**LEGEND**	*Confirmatory age effect	**Confirmatory age effect					
			**New genes for stroke**	**Aged-specific 3 days**	**Aged-specific 14 days**	**Young-specific, 3 days**	**Young-specific, 14 days**

*There were several distinct patterns of gene regulation: persistently upregulated (A), transiently upregulated, (B), “late-upregulated” (C), or “late-downregulated” (D). Aged animals showed larger numbers of genes that were late-upregulated (type C). The young rats, in contrast, had a much larger number of transiently upregulated (type B). Note that this representation does not take into account the fold changes for individual genes but the relative change in gene expression at days 3 and 14 post-stroke. FC, fold change; SD, standard deviation; EP, expression pattern; NC, no change; TF, transcription factor; EC, endothelial cell; BV, blood vessel; VSMC, vascular smooth muscle cell; PVC, perivascular cells; BL, basal lamina; ECM, extracellular matrix*.

### Persistent upregulation of matrix proteases and inflammatory mediators in the brains of aged rats

Genes that were new for stroke or showed an age-dependent expression were further verified by RT-PCR (Table 2). An early event after an ischemic insult is the activation of microglia in the peri-infarcted area, especially in aged rats (Popa-Wagner et al., 2007a).

Activated microglia and macrophages release the pro-inflammatory cytokine tumor necrosis factor alpha (TNF alpha) into the infarct area, which induces a number of pro-inflammatory changes; these in turn increase leukocyte adhesion, transendothelial migration, and vascular leakage and edema formation (Bradley, 2008). TNF-alpha is synthesized as a trimeric type II transmembrane precursor, which is then converted to a soluble form by the sheddase activity of ADAM17 (TACE-tumor necrosis factor alpha converting enzyme) a membrane-anchored metalloproteinase on EC and pericytes that was upregulated in both age groups, with higher expression in aged brains (twofold vs. young brains). *Adam 17* then stimulates *Notch* signaling and *TNF-alpha* expression, two potent promoters of angiogenesis (Gridley, 2007; Bradley, 2008; Chen et al., 2009). ADAM17 is further responsible for the proteolytic release of the vascular endothelial growth factor (VEGF) receptor 2 (VEGF2) (Swendeman et al., 2008; Weskamp et al., 2010).

TNF alpha is also a potent inducer of angiogenesis via VEGF, and two genes of the TNF family, *Tnfrs1a* and *Tnfrs1b*, were belatedly upregulated (twofold over the levels shown by the young animals) in the lesioned area of aged rats. Of these, *Tnfrs1b* has not been previously described in post-stroke angiogenesis. The pro-inflammatory *TNF alpha* also caused a twofold higher induction of the cytokine *Cxcl12* mRNA by day 14 in the brains of aged vs. young rats.

Disruption of EC contact and their detachment from the basal lamina (BL) are caused by the family of matrix metalloproteases (MMPs) during the tissue remodeling process (Sabeh et al., 2009; Liu et al., 2010). A new player in inflammation-induced vascular remodeling is collagenase *Mmp8*, which is produced by macrophages, and which is abundantly expressed in the infarcted area. Of note was the persistent upregulation of the *Mmp12* gene encoding a metalloelastase and *Mmp14* encoding a collagenase secreted by fibroblasts in the infarcted area of aged rats at 2 weeks post-stroke. We also noted the downregulation of *Bai2*, a brain-specific, *de novo* angiogenesis inhibitor (Table 2).

### Post-stroke sprouting angiogenesis is delayed in aged brains

Hypoxia is a potent stimulator of angiogenesis. Among “new-for-stroke,” pro-angiogenic transcription factors, we noted the constant and robust upregulation (fourfold to sixfold) of hematopoietically expressed homeobox (*Hhex*), runt-related transcription factor 1 (*Runx1*), and the SRY-box containing gene 7 (*Sox7*), all of them previously unknown to stroke models.

Tissue hypoxia activates *Vegfa* and *Vegfc* expression as well as the expression of other transcripts encoding angiogenic factors such as angiopoietin-2 (*Angpt2*) and the “new-for-stroke,” angiopoietin-like 2 (*Angptl2*), angiopoietin-like 4 (*Angptl4*), and the endothelial and SMC chemoattractant *Cxcl1* and its receptor *Cxcr2*, on EC. We noted the mirrored expression of *Angpt2* and *Angptl4*, those expression decreased with time in young rats but increased by day 14 in the brains of aged rats (Table 2).

Vascular endothelial growth factor-A and its receptors VEGFR1, VEGFR2, and neuropilins (NRP1, NRP2) play critical roles in angiogenesis. Of note was the specific and delayed upregulation (5.7-fold) of *Nrp1* mRNA in the peri-infarcted area of aged rats.

VEGFR2-induced migration of EC is modulated by several genes including calcium and integrin-binding 1 (calmyrin)(*Cib1*), collagen, type VIII, alpha 1 (*Col8a1*), endoglin (CD105)(*Eng*), fibulin-5 (*Fbln5*), integrin, alpha 5 (fibronectin receptor) (*Itga5*), lymphoid enhancer-binding factor 1 (*Lef1*), platelet/EC adhesion molecule 1 (*Pecam1*), pituitary tumor-transforming 1 interacting protein (*Pttg1ip*), S100 calcium-binding protein A4 (*S100a4*), SH2B adaptor protein 3 (*Sh2b3*), tropomyosin 3, gamma (*Tpm3*), tropomyosin 4 (*Tpm4)*. Except for *Fbln* and *Sh2b3*, all other transcripts showed delayed upregulation to various levels (threefold to eightfold) in the brains of aged rats. Most of them, except *Eng* and *Pecam1*, have not been previously described in stroke models or human cases. Of note was the consistently higher expression of *Plod2* and *S100a4* mRNAs, two markers of proliferating fibroblasts (Basile et al., 2011) in young animals (Table 2).

The proliferation of vascular SMCs, the second major cell type involved in post-stroke angiogenesis, was modulated by several “new-for-stroke” genes including the pro-angiogenic factors lymphoid enhancer-binding factor 1 (*Lef1*), transgelin (*Tagln*), wingless-type MMTV integration site family, member 4 (*Wnt4a*) but also by the well-known matrix metallopeptidase 2 (*Mmp2*). In addition, *Lef1* and *Wtn4a* expression showed a strong age effect, being increased in young animals only. In contrast, in aged animals *Lef1* and *Wtn4* transcripts were increased with delay by twofold to threefold over the levels in young animals. Similarly, *Vegfa* and *Vegfc* mRNAs were increased at day 14 after stroke only in young animals albeit at moderate levels (about twofold) (Table 2).

Hypoxia-induced vessel dilation leads to the release of pericytes, SMCs, and fibroblasts. During the activation phase of angiogenesis, ECs and fibroblasts migrate into extracellular space, proliferate, and release cytokines and angiogenic chemokines like CXCL1, CXCL12/CXCR4, and VEGF, which in turn activate circulating EPCs, which eventually integrate into tube structures and differentiate into mature EC that become incorporated into the newly formed blood vessels (Kanzler et al., 2013). The high *Cxcl1* mRNA levels in young but not aged rats at day 3 post-stroke suggest that the recruitment of EPCs may be more efficient in young animals. CXCL12 and its receptor CXC chemokine receptor 4 (CXCR4), which are expressed at high levels in both vascular and hematopoietic progenitor cells, play a critical role in the development of the heart and blood vessels, as well as in the regulation of motility and differentiation in EPC via the Rho GTPase, *Rac2* (Shen et al., 2012) (Table 2).

### Reconstruction of a new basal lamina and ECM occurs earlier in young brains

During the resolution phase, migration and proliferation of ECs is stopped, and at this step ECs reconstitute their BL. Except for *Col6a3* mRNA, which showed constant high levels in both age groups, the expression of collagen type IV, alpha 2 (*Col4a2*), fibronectin 1 (*Fn1*), laminin gamma 1 (*Lamc1*), nidogen 2 (*Nid2*), and podoplanin 3 (*Plod 3*) mRNAs was higher in the brains of young animals at day 3 post-stroke as compared to aged animals, strongly suggesting that BL and extracellular matrix (ECM) reconstruction occurs earlier in young animals. Nidogen 2 is a ubiquitous component of blood vessel BL. The nidogen–laminin γ1 interaction plays a key role in BL assembly (Mokkapati et al., 2011). New genes to be considered for vascular restoration after stroke included *Col6a3, Fn1*, and *Lamc1* (Table 2).

### Disorganized tube formation and maturation in aged rat brains

Subsequent to EC and EPC proliferation, the new cells reorganize into three-dimensionally capillary-like tubular structures. Under hypoxic conditions, ANG-2 promotes the dissociation of pericytes from pre-existing vessels and primes the vasculature for an angiogenic response by increasing vascular permeability to proteases, inflammatory cytokines, or angiogenic myeloid cells (Hammes et al., 2004). During BV maturation, ANGPT2–TIE2 induces EC apoptosis and participates, along with VEGF-C in lymphatic patterning. For *Angpt2* mRNA, we noted a mirrored expression in the two age groups: the *Angpt2* mRNA decreased from day 3 to day 14 in the young rats but then increased during the same time period in aged rats.

Vascular maturation also involves endothelial cell apoptosis. Pro-apoptotic factors and inducers of apoptosis in EC included the tumor necrosis factor, member 10 (*Tnfsf10*) mRNA, which showed very high levels by day 14 in aged rats as compared to young rats, highly suggestive of a premature depletion of ECs that are needed for vascular reconstruction (Chiang et al., 2013). Likewise, we noted the low expression levels for the runt-related transcription factor 3 (*Runx3*) mRNA, a negative regulator of EPC proliferation, especially in aged animals.

Quiescent ECs are covered by pericytes that secrete ANG-1, which contributes to vessel stabilization by facilitating EC–ECM and EC–mural cell interactions. Hypoxia, VEGF-A, and PDGF-B have been reported to elevate ANG-1 expression in perivascular cells such as pericytes or SMCs (Park et al., 2003). A delayed upregulation in the brains of aged rats was also recorded for angiopoietin-1 (*Angp1*) mRNA, mainly expressed in perivascular cells (pericytes), collagen type III (*Col3a1*) mRNA, a component of adventitia secreted by fibroblasts, and *Tnfsf10* transcripts.

A timely expression in both age groups was noted for collagen type I (*Col1a2*) mRNA, a component of the adventitia secreted by fibroblasts, integrin beta 4 (*Itgb4*), fibronectin type III domain containing 1 (*Fndc1*), the pro-angiogenic *Notch2*, procollagen C-endopeptidase enhancer (*Pcolce*), serine carboxypeptidase 1 (*Scpep1*), and *Tie1 mRNAs*, which encodes a receptor on EC involved in capillary maintenance by inhibiting the Angpt1/Tie signaling pathway (Yuan et al., 2007).

An *untimely* expression was seen for insulin-like growth factor binding protein 7 (*Igfbp7*) and Glypican-3 (*Gpc3*) mRNAs. *Gpc3* encodes the co-receptor for fibroblast growth factor-9 (FGF9) and a loss of glypican-3 function causes growth factor-dependent defects in cardiac and coronary vascular development (Ng et al., 2009). Both transcripts were robustly increased (fourfold to sixfold) by day 14 in aged animals, suggesting that angiogenesis was just being initiated at this timepoint rather than entering the resolution phase, as was the case in young rats (Table 2).

### Blood vessel stabilization and fibrosis

Genes contributing to BV stabilization were numerous. Of these, we noted the timely and highly expressed (fourfold to eightfold) cysteine-rich (osteonectin) (SPARC), especially in aged rats (twofold over young animals).

However, excessive synthesis of ECM and BL components may lead to fibrosis, a process that is greatly augmented by the presence of myofibroblasts in the inflammatory milieu of post-stroke aged animals. Likewise, an inhibition of metalloproteinases may have the same fibrogenic effect by allowing the accumulation of ECM components. Tissue inhibitor of metalloproteases (*Timp1)*, which encodes a natural inhibitor of matrix proteases, was greatly increased (31-fold) by day 14 in aged but not young rats. Likewise, interleukin 13 receptor, alpha 1 (*Il13ra1*) gene encoding a receptor on SMCs and fibroblasts and which is also a new player for stroke scholars, may promote fibrosis by inhibiting metalloprotease activity in fibroblasts (Bailey et al., 2012). Metalloproteases such as MMP2 also contribute to fibrosis directly as modulators of the conversion of fibroblasts to myofibroblasts, especially in aged rats. Indeed, *Mmp2* expression was greatly increased by day 14 in aged rats (sevenfold over the young rats).

Post-stroke inflammation is a major factor that augments the fibrosis. During the resolution phase at day 14, we noted a large increase in the expression of inflammation-related genes required for fibrosis in aged rat brains. The inflamed endothelium expresses large amounts of transcripts (sixfold over the young rats), encoding the angiogenic stromal cell-derived factor-1a CXCL12, a chemoattractant for EPCs and SMCs. Activated EPCs upregulate gene expression for chemokine receptors, *Cxcr2* and *Cxcr4*, the principal receptors for CXCL1 and CXCL12, respectively (Kanzler et al., 2013).

Macrophages are a critical factor in the initiation of fibrosis through the production of TNF, TGFβ, IL-18, and of PDGF (Wynn, 2008). Macrophage expressed 1 (*Mpeg1*) mRNA showed large and persistent increase in both age groups. Along this line of evidence, IL-18 after stroke is produced by microglia and contributes to fibrosis in the aged rat brain via STAT3 activation, leading to a large accumulation (237-fold over controls) of collagen III (*Col3a1*) transcripts that are normally expressed in adventitial fibroblasts (Matsui et al., 2013) (Table 2).

TGF-beta signaling is the second major fibrogenic pathway in the post-stroke brain. *Tgfb1* and *Tgfbr2* mRNAs showed extremely high levels (10- to 57-fold over controls) at day 14 in post-stroke rats. *Tgfbi*, which is new-for-stroke research, was, to the contrary, specifically increased in young animals early after stroke and recovered by day 14 post-stroke. Aged animals failed to upregulate *Tgfbi* transcripts. However, other components of the TGF-beta signaling such as TGF beta, encoding receptor I (*Tgfbr1*) did not show an age-dependency.

TGF-beta signaling together with PDGF induces the proliferation of myofibroblasts, which secrete collagen type I (*COL1a1*), collagen triple helix repeat containing 1 (*CTHR1*) and prolyl 4-hydroxylase, beta polypeptide (*P4Hb*), those mRNAs were increased with delay at day 14 in the brains of aged animals. In particular, we noted the high *Cthrc1* transcript levels specifically in the aged animals (sixfold over the young animals). CTHRC1 is expressed by injured arteries, where it is induced abundantly in adventitial fibroblasts and neointimal SMCs and may inhibit SMC proliferation via the Tgf β pathway (LeClair et al., 2007). However, both *Pdgfra* and *Pdgfrb* transcripts encoding the PDGF receptors on pericytes, had a regular expression time course in both age groups. Of note was the expression of *Wnt5a*, which showed consistently increased levels only in aged animals (Table 2).

Post-stroke angiogenesis resembles in many ways the angiogenesis in tumors, i.e., the newly formed vessels are leaky via the Il6r/Il6st/STAT1/3 pathway. IL-6 is synthesized by myeloid cells, fibroblasts, and ECs and the binding to its receptor (IL6R) that triggers gp130-mediated activation of STAT1 and STAT3, was found to be involved in vascular leakage (Wei et al., 2013). *Stat1* and *Stat2* are new players in the stroke search for novel therapeutic targets. Of note, *Stat2* expression was increased by day 14 only in the aged animals.

## Histology

**Figure 2:**
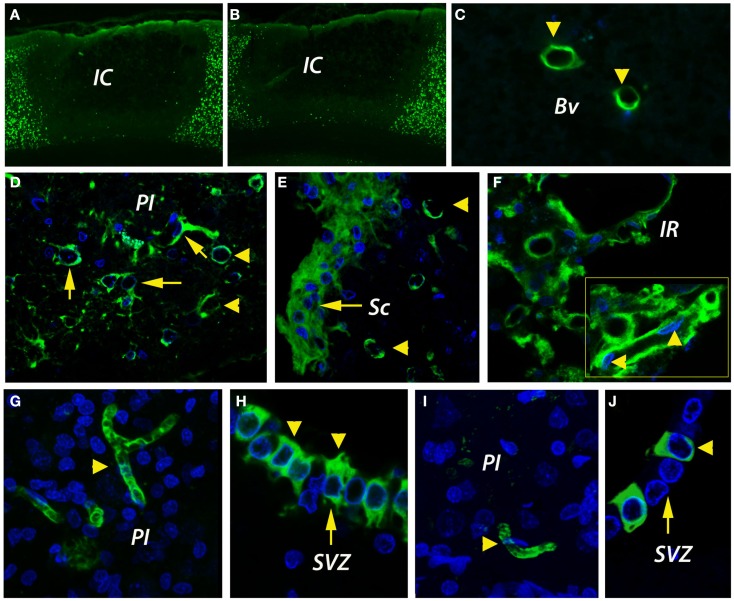
**Infarct size and angiogenesis assessment by immunofluorescence**. **(A,B)** By NeuN immunohistochemistry, the cortical infarcts were similar in size in both age groups, i.e., 35 ± 3.1% of total cortical volume in young rats and 40 ± 7.9% in aged rats. **(C)** In the unlesioned hemisphere, CD31-positive blood vessels (green) were rarely detected. **(D)** By day 14, new blood vessels were emerging in the peri-infarcted area of young animals. The revascularization process in aged animals was delayed and was characterized by the accumulation of CD31-immunopositivity in the fibrotic scar **(E)**. Beyond the fibrotic scar, however, in a region that we dubbed “islet of regeneration,” vigorous angiogenesis was detected in aged animals as well **(F)**. By day 14, mature BV had developed in the peri-infarcted area of young animals **(G)**. Similarly, new, mature blood vessels were detected in the same areas of the aged rats **(I)**. Please note the unexpected expression of CD31 in the wall of the subventricular zone both in young **(H)** and aged animals **(J)**. **(C–J)** are *Z*-projection images. BV, blood vessel; IC, infarct core; IR, islet of regeneration; PI, peri-infarct; Sc, scar; SVZ, subventricular zone.

The development of the infarct core was visualized by immunohistochemistry for NeuN, a sensitive indicator of neuronal viability. As previously reported for this model (Popa-Wagner et al., 2007b), the development of the infarct was more rapid in aged rats, but by day 14, the cortical infarcts were similar in size in both age groups, i.e., 35 ± 3.1% of total cortical volume in young rats and 40 ± 7.9% in aged rats (Figures 2A,B).

### Angiogenesis assessment by immunofluorescence

CD31 (PECAM-1) antigen is expressed on ECs and pericytes, and is considered a marker of post-stroke angiogenesis (Deddens et al., 2013). In the unlesioned hemisphere, blood vessels were rarely detected by CD31 immunofluorescence (Figure 2C). By day 14 after stroke, new blood vessels were emerging in the per-infarcted area of young animals (Figure 2D). The revascularization process in aged animals was delayed and was characterized by the accumulation of CD31-immunopositivity in the fibrotic scar (Figure 2E). Beyond the fibrotic scar, however, in a region that we dubbed “islet of regeneration,” vigorous angiogenesis was detected in aged animals as well (Figure 2F). By day 14, mature BV had developed in the peri-infarcted area of young animals (Figure 2G). Similarly, new, mature blood vessels were detected in the same areas of the aged rats (Figure 2I). Quite interestingly, we report the unexpected expression of CD31 in the wall of the subventricular zone (SVZ) both in young (Figure 2H) and aged animals (Figure 2J).

### Fibroblasts may contribute to the fibrotic scar

**Figure 3:**
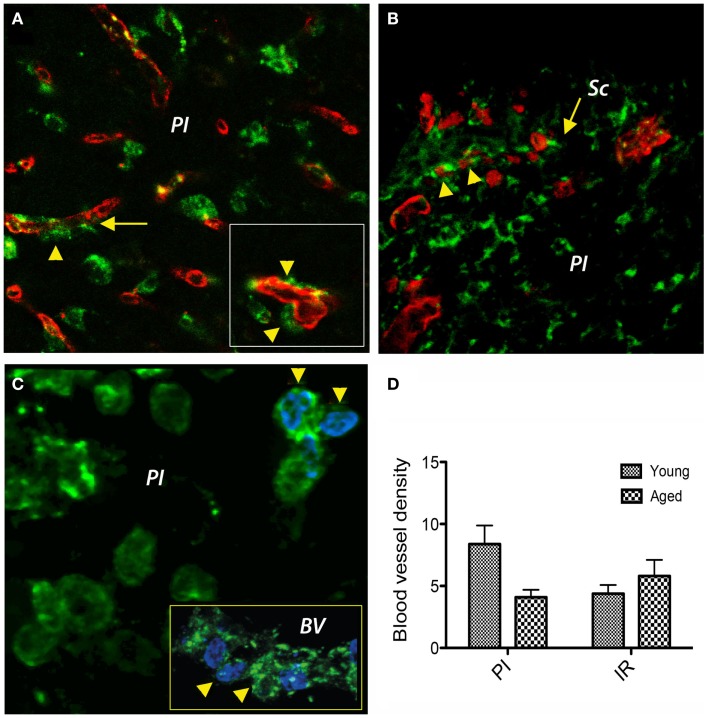
**Double immunofluorescence prolyl 4-hydroxylase/CD31 and prolyl 4-hydroxylase/BrdU in the peri-infarcted area**. **(A)** Double labeling of cells with a P4Hbeta antibody (green) and CD31 (red) revealed that under hypoxic conditions P4Hb-positive fibroblasts/fibrocytes seems to emanate from the vascular wall (inset, arrowheads) a process that was more evident in young brains. **(B)** In the aged animals, we noted a preferential agglomeration/accumulation of fibroblasts in the scar region. **(C)** Double labeling of cells with a P4Hb (green) and the proliferation marker BrdU (blue) in aged rat brains revealed that some proliferating fibrocytes/fibroblasts had BrdU-positive nuclei in the peri-lesional area (arrowheads). Eventually, some co-labeled cells emanated from the capillary wall [**(C)**, inset, arrowheads]. **(D)** At day 14 post-stroke, the vascular density was higher in the peri-infarcted area of young animals as compared to the similar region of aged rats (twofold). However, beyond the inhibitory fibrotic scar, the vascular density was similar in the two age groups. **(A–C)** represent *Z*-projection images.

Under hypoxic conditions, the newly generated fibroblasts express prolyl 4-hydroxylase (P4Hbeta), a member of the non-heme iron (II), α-ketoglutarate-dependent dioxygenase family, which catalyzes hydroxylation of proline residues in the collagen strands. The fibroblasts/fibrocytes emanate from the CD31-positive vascular wall, a process that was more evident in young brains (Figure 3A). However, in the aged animals we noted a preferential agglomeration/accumulation of fibroblasts in the scar region (Figure 3B). Double labeling of cells with a P4Hbeta antibody (green) and a proliferation marker (BrdU, blue) in aged rat brains revealed that some proliferating fibrocytes/fibroblasts had BrdU-positive nuclei in the peri-lesional area (arrowheads). Eventually, some co-labeled cells emanate from the capillary wall (Figure 3C, inset, arrowheads). Quantitatively, at day 14 post-stroke the vascular density was higher in the peri-infarcted area of young animals as compared to the similar region of aged rats (twofold). However, beyond the inhibitory fibrotic scar, in a region made of soft tissue that we dubbed “islet of regeneration,” the vascular density was similar in the two age groups (Figure 3D).

### Semi-quantitative analysis of angiogenesis in the rat model

**Figure 4:**
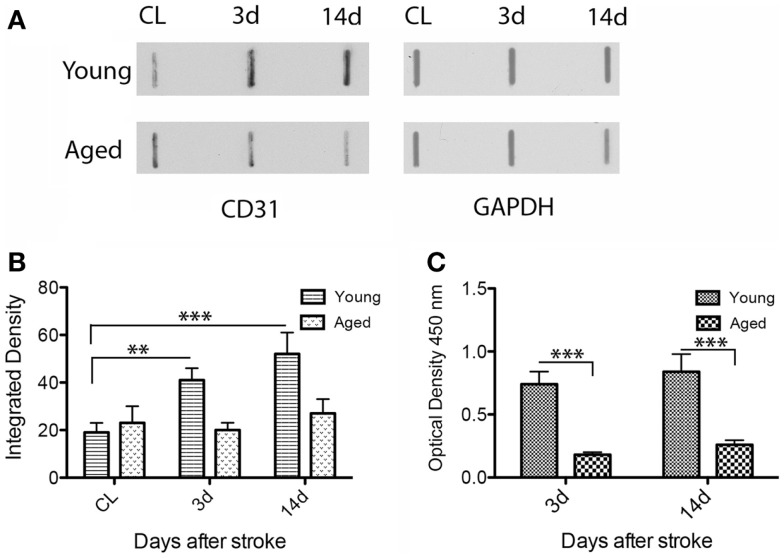
**Quantitative analysis of CD31 expression in the rat model**. By dot blot **(A)** and ELISA analysis **(C)**, CD31 immunoreactivity in the peri-infarcted area was slightly increased from day 3 to day 14 in both age groups. However, CD31 levels in the young animals were clearly higher than those in aged animals both at day 3 (fourfold; *p* = 0.001) and day 14 (threefold; *p* = 0.001) both by dot blot **(B)** and ELISA**(C)**.

By dot blot (Figures 4A,B) and ELISA analysis, CD31 immunoreactivity in homogenates of the peri-infarcted cortex was slightly increased from day 3 to day 14 in both age groups (Figure 4A). However, by ELISA, the CD31 levels in young animals were much higher than those of aged animals both at day 3 (fourfold; *p* = 0.001) and day 14 (threefold; *p* = 0.001) (Figure 4C).

### Angiogenesis in post-stroke human tissue

**Figure 5:**
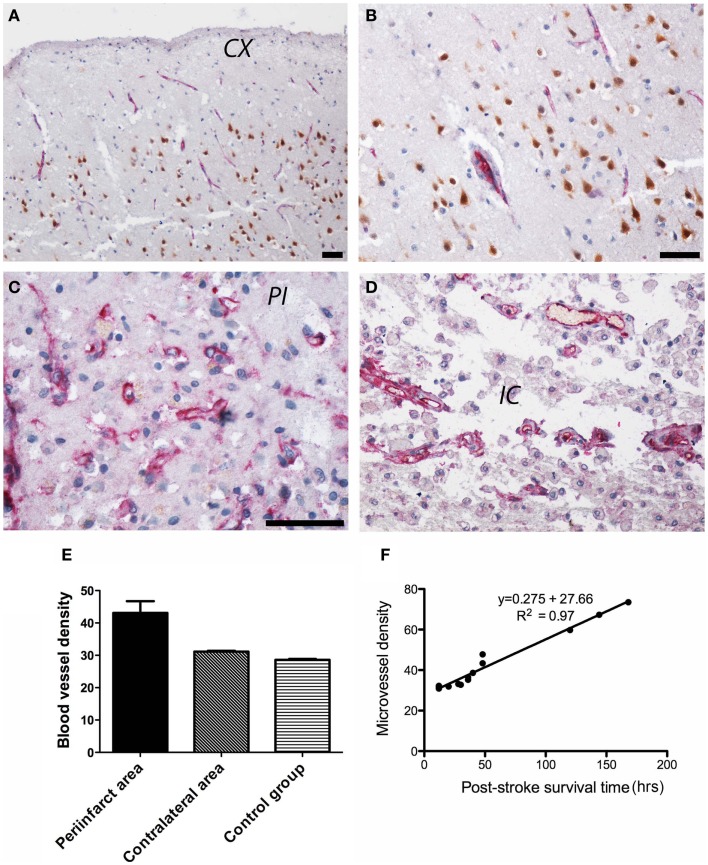
**Angiogenesis in post-stroke human tissue**. **(A)** In control subjects, CD105 immunoreactivity (red) was present in the endothelial lining of the cortical capillaries in an orderly pattern. Neuronal nuclei are shown in brown. An enlarged view is shown in **(B)**. **(C)** In the peri-infarcted area, CD105 immunostaining was present in tortuous, branched blood vessels including randomly scattered ECs. **(D)** In the infarct core, CD105 immunopositivity was present in the endothelium of spared blood vessels. **(E)** Quantitatively, the highest vascular density (given as number of microvessels/0.7386 mm^2^) was in the peri-infarcted area as compared to the contralateral side and the control group. **(F)** We also noted a strong correlation between post-stroke angiogenesis and post-stroke survival time [*r*(13) = 0.984, *p* < 0.001]. CX, cortex; PI, peri-infarct; IC, infarct core. Bars, 50 μm.

In control subjects, CD105 immunoreactivity was detected in the endothelial lining of the cortical capillaries in an orderly pattern (Figures 5A,B). In the peri-infarcted area, CD105 immunostaining was present in an angiogenic complex consisting of numerous tortuous, branched blood vessels of various lengths including randomly scattered ECs (Figure 5C). In the infarct core, CD105 immunopositivity was present in the endothelium of spared blood vessels (Figure 5D). The angiogenic process was mostly evident in the peri-infarcted area, which showed the highest vascular density as compared to the contralateral side and the control group (Figure 5E). We also noted a strong correlation between post-stroke angiogenesis and post-stroke survival time [*r*(13) = 0.984, *p* < 0.001] (Figure 5F).

### Patterns of gene expression after stroke in animal model

**Figure 6:**
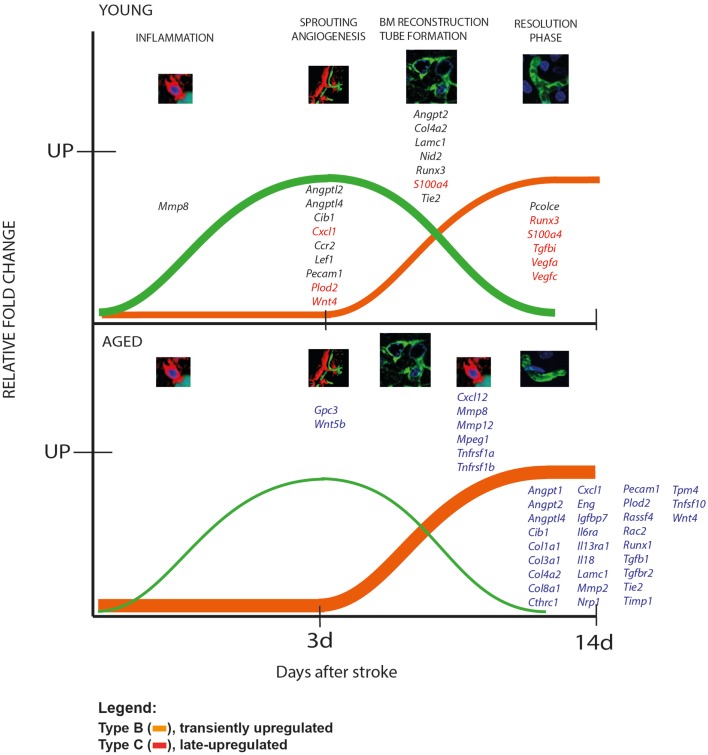
**Patterns of gene expression after stroke in animal model**. We distinguished two patterns of gene regulation, transiently upregulated (type B, expression recovered after the acute phase, green line) and “late-upregulated” (type C, expression went up after the acute phase; orange line). Please note that aged animals showed larger numbers of genes that were belatedly upregulated than the young animals. The young rats, in contrast, had a larger number of transiently upregulated including *Cxcl1, S100a4, Runx3, Tgfbi, Vegfa, Vegfc*.

The kinetics of gene expression over a longer time period gives us clues as to what processes in the long run could be defective at the level of transcription in aged rodents. We distinguished two patterns of gene regulation, as depicted schematically in Figure 6 and shown in detail in Table 2: transiently upregulated (type B, expression recovered after the acute phase) and “late-upregulated” (type C, expression went up after the acute phase).

A comparison of the global pattern of gene expression indicated that aged animals showed a larger numbers of genes that were belatedly upregulated (orange line) than the young animals. The young rats, in contrast, had a larger number of transiently upregulated genes (green line) (Figure 6, Table 2).

## Discussion

Stroke induces a specific remodeling of the brain vasculature. Using an aged rat model of stroke, we found that at 2 weeks after stroke the microvascular density was reduced in aged rats as compared to young animals on a background of persistent upregulation of genes coding for matrix proteases and inflammatory mediators. However, beyond the inhibitory fibrotic scar, in a region made of soft tissue that we dubbed “islet of regeneration,” the vascular density was similar in the two age groups. Unlike in rats, the post-stroke angiogenesis in human patients is vigorous at 1 week post-stroke, and correlates well with the post-stroke survival time. By comparative transcriptomics of angiogenesis, we identified 36 new stroke-related genes some of which may be used as new therapeutic targets that may help redress the dysregulation of *angiogenesis* in the infarcted area of aged brain. We also found that the aged human brain is capable of mounting a vigorous angiogenic response after stroke, which most likely reflects the remaining brain plasticity of the aged brain.

### Sprouting angiogenesis is impaired in aged rats

Angiogenesis has multiple beneficial roles in the post-ischemic brain, contributing to (i) the stabilization of brain hemodynamics, thus preventing subsequent stroke events, (ii) the prevention of neuronal degeneration via release of neurotrophic growth factors, (iii) the removal of dead cells from the tissue by blood-derived macrophages that find access to the ischemic lesion, and (iv) the attraction of neural precursor cells that again release growth factors (Slevin et al., 2006; Hermann and Zechariah, 2009).

Although infarct development was more rapid in aged rats by day 14, the cortical infarcts were similar in size in both age groups (Popa-Wagner et al., 2007a). Unlike infarct size, sprouting angiogenesis was delayed in aged animals. The young animals had higher levels of CD31 and a significantly increased vascular density. The disorganized and delayed vascular tube formation in aged rats corresponded to a divergent pattern of gene expression by day 14 in the infarcted aged brains as compared to the young animals. A similar divergent pattern of gene expression has been reported by our group for genes involved in CNS physiology and homeostasis, or for genes required for embryonic development and CNS remodeling after stroke (Buga et al., 2012).

Automatic assignment of genes to specific processes is a valuable technique, but may nevertheless lead to spurious functional associations. It is thus important to supplement these methods with one-by-one gene function searches in the context of the pathophysiology of stroke. Because young rats recover behaviorally within 2 weeks after stroke (Buchhold et al., 2007), in this Section “Discussion” we assume that the gene expression related to *angiogenesis* is optimal in the young animals. The transcriptomic analysis strongly suggests that the persistent upregulation of several genes involved in matrix degradation and inflammation is mainly responsible for the delayed angiogenesis in the infarcted area of aged rats. In response to prolonged hypoxia, fibroblasts degrade type I collagen, one of the major extracellular proteins of the vasculature, and the available evidence suggests that type I collagenolysis is mediated by secreted as well as membrane-anchored members of the matrix metalloproteinase (MMP) gene family, including *Mmp8, Mmp12*, and *Mmp14* (Sabeh et al., 2009; Liu et al., 2010). In young animals, the MMPs are strongly upregulated early after stroke, but then decrease or remain at the same level by day 14 post-stroke. In aged animals, in contrast, *Mmp8, Mmp12*, and *Mmp14* were increased by day 14. Similar increases were noted for macrophage marker Mpeg1 and monocyte recruitment factor, *Tnfrsf1a* and *Tnfrsf1b* transcripts.

Matrix metalloproteinases are responsible for the breakdown of the ECM under hypoxic conditions. During the recovery phase, however, MMP14 could play an important pro-angiogenic role and promote tissue recovery after stroke. Indeed, impaired angiogenesis has been also observed in Mmp14 null mice (Shimokawa, 2008). Nonetheless, persistent overexpression of MMPs might lead to inefficient angiogenesis and tissue repair after stroke (Danielsen et al., 2011). Hyperactivation of MMP14 has been associated with increased leakiness and an immature blood vessel phenotype (Sounni et al., 2010), but also with formation of fibrous tissue by promoting collagen invasion in vascular SMCs (Mountain et al., 2012). Likewise, in the latest stage after ischemia, MMP14 is responsible for chemokine activation, in particular TGF-beta pathway via ALK5/Smad3 (Sounni et al., 2010), which can affect the stability of blood vessels.

In previous work, we described the fulminant development of inflammation in the infarcted area of aged rats (Badan et al., 2003). Prolonged hypoxia induces blood vessel dilation, detachment of pericytes, and EC sprouting. When vessels lose pericytes, they become hemorrhagic and hyperdilated, which leads to edema (the main cause of death in the first 3 days post-stroke) and facilitates the infiltration of immune cells into the lesioned area.

TNF-alpha is a potent neuromodulator with multiple effector functions, and has been strongly implicated in the recruitment of leukocytes and monocytes after injuries both centrally and in the periphery via TNFR1, which is encoded by the *Tnfrsf1a* and *Tnfrsf1b* genes. TNF receptors are expressed on many cell types including ECs of fibrovascular tissue (Majka et al., 2002), and promote inflammation through activation of cytokines and MMPs (Bradley, 2008; D’Mello et al., 2009; Jasielska et al., 2010). Many of the pro-inflammatory responses of TNF *in vivo* can be traced to its effects on the vascular endothelium and leukocyte and monocyte infiltration. TNF induces the expression of endothelial adhesion molecules that support leukocyte–endothelial interactions and stimulates the local production of chemokines like CCLl2 and CXCL10, which promote leukocyte activation and transmigration into tissue (Bradley, 2008). Our results indicate that *Tnfrsf1a* and *Tnfrsf1b* mRNAs are robustly upregulated in the early stage of ischemic damage in both age groups, but its expression was robustly increased by day 14 post-stroke in the aged brains only. Recent studies with *Tnfrsf1a*^−/−^ and *Tnfrsf1b*^−/−^ mice have shown that *Tnfrsf1b* promotes the recruitment of inflammatory cells to the site of injury and exacerbated pathologic angiogenesis, whereas *Tnfrsf1a* was required for apoptosis, suggesting persistent inflammation and apoptotic events in the aged brains.

A sustained expression of the pro-inflammatory TNF is also responsible for prolonged upregulation of CXCL12–CXCR4 chemokine ligand–receptor axis on infiltrating leukocytes in the aged brains (Wang et al., 2012). Soon after stroke *Cxcl12* expression promotes EPC recruitment at the injury site, but later on it becomes a powerful chemoattractant for SMCs that may further contribute to the formation of the fibrotic scar (see below).

### Angiogenesis is increased at 2,weeks post-stroke in young vs. aged rats

Following stroke, angiogenesis, which is defined as formation of microvessels from existing microvessels, is enhanced in the per-infarcted region as compared to the contralateral unlesioned cortex (Wang et al., 2005; Kilic et al., 2006; Zechariah et al., 2013). By CD31 immunohistochemistry and ELISA assay, the concentration of CD31 and the density of newly formed blood vessels were significantly higher at day 14 in the brains of post-stroke young animals. Increased angiogenesis in post-stroke young animals was presumably due to increased sprouting angiogenesis. Remarkable was the delayed angiogenic response on a background of a fulminant inflammatory reaction in the aged, injured brains.

Angiogenesis in young animals benefited from the early, vigorous expression of the angiogenic factors *Angpt2, Angptl2, Angptl4*, and *Pecam* (*CD31*) in EC (Le Jan et al., 2003; Tazume et al., 2012) as well as an increased expression of *Cxcl1* mRNA in ECs. The angiogenic chemokine CXCL1 is a secreted growth factor that interacts with the G-protein-coupled receptor CCR2 on ECs (Miyake et al., 2013). ANGPTL2 induces sprouting in ECs through an autocrine or paracrine action and therefore is a potent stimulator of angiogenesis (Kim et al., 1999). Likewise, the young rats benefited from the early upregulation of genes required for endothelial cell proliferation and migration, including *Cib1* and *Lef1* mRNAs. CIB1 is a calcium and integrin-binding protein-1 that is essential for proper EC signaling and function such as migration, proliferation, and nascent tubule formation, and therefore is critical in ischemia-induced angiogenesis in the retina, as well as ischemia-induced adaptive angiogenesis (Zayed et al., 2007).

Sprouting angiogenesis was delayed in injured, aged rat brains. In particular, we noted the mirrored expression of *Angptl4, Cib1* and *Col8a1, Eng, Lef1, Nrp1, Pecam, Tpm4* transcripts, suggesting that delayed cellular proliferation in conjunction with a delayed secretion of proper migrating substrates is the main cause of delayed sprouting angiogenesis in the aged rat brains after stroke. The normal vessel wall ECM is composed predominantly of type I and III collagens, elastin, and proteoglycans, with smaller amounts of fibronectin, laminins, and type IV collagen.

Regarding with signaling pathways, those expressed during embryogenesis such as FGF/FGFR, Notch, Shh, or Wnt7a/7b are not activated during post-stroke angiogenesis suggesting that post-stroke angiogenesis is not simply a recapitulation of developmental angiogenesis. One of the few activated signaling pathways was Wnt4. In response to arterial injury, SMCs shift from a quiescent, contractile phenotype to a proliferative, synthetic state. In this phenotypic transition, *Wnt4a* and *Lef1* act in concert to promote endothelial cell proliferation. Lymphoid enhancer-binding factor 1 (*Lef1*) is a member of the Lef/Tcf transcriptional regulator family that mediates beta-catenin-dependent transcription in the Wnt-beta-catenin pathway and promotes endothelial cell migration (Planutiene et al., 2011). Aged animals failed to initiate the expression of this EC migration pathway and displayed abnormally high levels of *Wnt4a* mRNA by day 14 post-stroke.

### Timely basal lamina reconstruction and tube formation in young brains

During the activation phase of angiogenesis, ECs and fibroblasts start to degrade their vascular BL, migrate into extracellular spaces, proliferate, and form vascular tubes. Delayed and persistent expression of the matrix-degrading proteases *Adam17* mRNA in aged brains may have delayed the reconstruction of BL in aged animals. Laminin gamma1 (*Lamc1*), *Nid2*, and *Plod2* mRNAs were increased exclusively in young animals in the early stage after cerebral ischemia, suggesting that these genes play an essential role in post-stroke angiogenesis. NID2 and LAMC1 are components of the BL and promote ECM interaction and cell adhesion. Likewise, PLOD2 is expressed in fibroblasts and is required for tube formation and ECM stiffening and collagen fiber alignment (Gilkes et al., 2013).

Angpt1/Tie2 signaling is mainly responsible for capillary maintenance. Targeted Tie1 disruption results in embryonic lethality due to edema, hemorrhage, and microvessel rupture, a finding that suggests that Tie 1 is required for vascular integrity (Yuan et al., 2007). An increased expression of *Angpt2, Col4a2, Runx3, S1004, Vegfa, Vegfc*, and *Tie2* mRNAs in the angiogenic area of young rats may have been also essential in promoting timely angiogenesis as compared to aged animals. *Vegfa* and *Vegfc* mRNAs were specifically increased by day 14 after stroke in young animals, and thus may have contributed to a sustained angiogenic response. This hypothesis is strongly supported by the mirrored expression of *Col4a2, Lamc1*, and *Tie2* in the angiogenic area of aged animals.

An increased collagen IV alpha2 level can act as negative regulator of angiogenesis in the latest stage after stroke by inhibiting angiogenesis via an integrin-mediated apoptotic mechanism (Kozulin et al., 2010). Collagen, type IV, alpha 2 (COL4A2) is an abundant component of BL of the cerebral vasculature and *Col4a2* mutations cause small-vessel disease and hemorrhagic stroke (Gould et al., 2006; Jeanne et al., 2012). Runx3 was one of the very few transcripts showing high levels at 2 weeks post-stroke in young rats. Runt-related transcription factor 3 (Runx3) is essential for normal development and may be required for maturation of newly formed blood vessels (Lee et al., 2013).

Vessel stabilization can be achieved by the upregulation of *Pdgfrb, S100a4, Scpep1*, and *SPARC* genes, which promote the recruitment of pericytes and SMCs on the emerging blood vessels. Fibroblast-derived proteins increase the stiffness of the matrix. One of those proteins, S100a4 (also called fibroblast-specific protein) promotes sprouting and lumen formation in three-dimensions. Our studies also suggest that the new “stroke genes” *Fnbl5, Fndc1, Pcolce, Scpep1*, and *Igfbp7* induce EC-lumen formation (Newman et al., 2011) by increasing the stiffness of the ECM and increasing BV elasticity (Pshezhetsky and Hinek, 2009). FBLN5 (fibulin-5) is an extracellular matrix calcium-binding glycoprotein expressed in elastic fiber-rich tissues that is necessary for elastogenesis (Yanagisawa et al., 2009; Noda et al., 2013).

Tie2 gene deletion causes embryonic death with endocardial defects, hemorrhaging, and impaired vascular network formation suggesting that Tie2 is primarily required for spouting angiogenesis and then for capillary maintenance (Puri et al., 1995; Patan, 1998). Tube formation and BV maturation was clearly delayed in the damaged area of aged animals and was due to the delayed upregulation of several key genes, including *Angpt1, Angpt2, Gpc3, Igfbp7, SPARC*, and *Tie2*.

We noted the unusually high levels of the pro-apoptotic gene *Tnfsf10* at day 14 in the peri-lesional area of aged brains, suggesting that the lower vascular density in the aged rat brains may be due to an increased apoptosis of EC.

### The buildup of fibrotic tissue was prominent in the scar region of aged rats

Persistent inflammation, excessive synthesis of the components of the BL as well as inhibition of matrix proteases may limit the plasticity and remodeling of blood vessels and favor the accumulation of fibrotic tissue in the scarring region after lesioning (Johnson and Dipietro, 2013). This phenomenon was exaggerated in aged rats by an increased expression of several new “stroke genes,” including *Il13a, Rassf4, Tgfbi*, and *Wnt5b*, in the peri-lesional cortex of aged rats only.

Following cardiac ischemia, it is currently thought that fibroblasts migrate to the infarcted region and, under the influence of TGF-beta, differentiate into a myofibroblast phenotype characterized by smooth muscle a-actin expression (Willems et al., 1994; Sun et al., 2000; Frangogiannis et al., 2002; Virag and Murry, 2003; Squires et al., 2005; Wynn, 2008). Our study suggests that a similar scenario may also hold for central nervous tissue. We found that P4hb-expressing fibroblasts displayed a preferential accumulation in the scar region of post-stroke aged brains, and may have contributed to the formation of the fibrotic scar via TGF-beta signaling. The source of migrating fibrocytes/fibroblasts appears to be the vascular wall itself, a phenomenon that we described for migrating pericytes after stroke in aged rats (Popa-Wagner et al., 2007b).

Previous studies have shown that TGF-beta-induced phenotypic switch of adventitial fibroblasts into hybrid non-muscle-SMC known as myofibroblasts is mediated by the MMP2/Timp1 pathway and occurs during wound healing and various fibrotic disorders (Short et al., 2004; Misra et al., 2010). During tissue healing, the primary role of scar myofibroblasts involves the synthesis and deposition of collagen α(1) type 1 and smooth muscle α-actin (El-Helou et al., 2012). Indeed, we noted extraordinarily high levels of *Col1a1, Mmp2*, and *Timp1* transcripts by day 14 in the peri-lesional area of the aged brains.

### The newly formed blood vessels may be leaky

Aberrant angiogenesis is a major contributor to tumor growth. There are parallels between the structure of post-stroke angiogenesis and cancer vasculature with regard to blood vessel immaturity and vascular permeability. An increased expression of the newly identified stroke genes *Il6ra, Il6st, S100a4, Stat1*, and *Stat2*, but also the known genes *Col1a1, Col1a2, Col3a1*, has been implicated in blood vessel remodeling and contributes to vascular rarefaction, leakage, and intracranial aneurysms (Peters et al., 2001; Basile et al., 2011).

In human aging brains, one of the most important changes is represented by a decrease of the normal cerebral vascular network. This may affect the ability of the aged subjects to mount an adequate pro-angiogenic response after stroke (Petcu et al., 2010). However, our findings indicate that aged human brains still have the capability to mount a vigorous angiogenic response, as measured by CD105 immunoreactivity, that correlated with the post-stroke survival time. However, we think that this correlation reflects the plasticity reserves of the aged brain rather than merely the angiogenic response.

### Robust angiogenesis beyond the fibrotic scar and the subventricular zone

The anti-angiogenic nature of the fibrotic scar in aged tissue is illustrated by the vigorous angiogenesis in the unstructured tissue of aged rats beyond the fibrotic scar (islet of regeneration). By analogy with “scar-free” healing of embryonic tissue (Ferguson and O’Kane, 2004), we hypothesize that the major “anti-angiogenic” factor is the precipitous and persistent post-stroke inflammation in the peri-infarcted area, below the fibrotic scar of the aged animals.

Also, a robust angiogenic response was observed in the SVZ. Angiogenesis beyond the fibrotic scar and in the SVZ has not been studied in detail. Recently, it was reported that platelet administration after stroke increases angiogenesis in the SVZ (Hayon et al., 2013).

## Conclusion

We found that both age groups initiated vigorous angiogenesis. However, the young rats had a higher vascular density by day 14. Key genes responsible for the increased vasculature density in young animals were *Cxcl1, Lamc1, Nid, Plod2, Wnt4*, and *Wnt5*, mostly required for sprouting angiogenesis and BL reconstruction. New genes for stroke also included *Angptl2, Angptl4, Mmp8, Mpeg1*, and *Tnfrsf1b*. The vast majority of genes showed delayed upregulated in the aged rats, presumably because of persistent post-stroke inflammation. The young rats, in contrast, had a much larger number of transiently upregulated genes. The angiogenic response in aged rats was further diminished by the buildup of fibrotic scar tissue. Beyond this barrier, however, the vascular density in aged brains was similar to that of the young animals. Similarly, we did not detect a decreased angiogenic response to stroke in the SVZ of the aged group. We also show that the aged human brain is still capable of mounting a vigorous angiogenic response after stroke that reflects, most likely, the remaining human brain plasticity (Mahncke et al., 2006; Mercado, 2008). Finally, some of the newly identified stroke-related genes may be used as new therapeutic targets that may help redress the dysregulation of *angiogenesis* in the infarcted area of aged brain.

## Conflict of Interest Statement

The authors declare that the research was conducted in the absence of any commercial or financial relationships that could be construed as a potential conflict of interest.

## Footnotes

^1^www.r-project.org

^2^www.bioconductor.org
